# A proliferative subtype of colorectal liver metastases exhibits hypersensitivity to cytotoxic chemotherapy

**DOI:** 10.1038/s41698-022-00318-z

**Published:** 2022-10-14

**Authors:** Liam F. Spurr, Carlos A. Martinez, Rohan R. Katipally, Soumya C. Iyer, Sian A. Pugh, John A. Bridgewater, John N. Primrose, Enric Domingo, Timothy S. Maughan, Michael I. D’Angelica, Mark Talamonti, Mitchell C. Posner, Philip P. Connell, Ralph R. Weichselbaum, Sean P. Pitroda

**Affiliations:** 1 Pritzker School of Medicine, The University of Chicago, Chicago, IL USA; 2 Department of Radiation and Cellular Oncology, The University of Chicago, Chicago, IL USA; 3 Ludwig Center for Metastasis Research, The University of Chicago, Chicago, IL USA; 4 Addenbrooke’s Hospital, Cambridge, UK; 5 UCL Cancer Institute, University College London, London, UK; 6 Department of Surgery, University of Southampton, Southampton, UK; 7 Department of Oncology, University of Oxford, Oxford, UK; 8 Department of Surgery, Memorial Sloan Kettering Cancer Center, New York, NY USA; 9 Department of Surgery, NorthShore University Hospital, Evanston, IL USA; 10 Department of Surgery, The University of Chicago, Chicago, IL USA

**Keywords:** Colorectal cancer, High-throughput screening, Cancer genomics

## Abstract

Personalized treatment approaches for patients with limited liver metastases from colorectal cancer are critically needed. By leveraging three large, independent cohorts of patients with colorectal liver metastases (*n* = 336), we found that a proliferative subtype associated with elevated CIN70 scores is linked to immune exclusion, increased metastatic proclivity, and inferior overall survival in colorectal liver metastases; however, high CIN70 scores generate a therapeutic vulnerability to DNA-damaging therapies leading to improved treatment responses. We propose CIN70 as a candidate biomarker to personalize systemic treatment options for patients with metastatic colorectal cancer. These findings are potentially broadly applicable to other human cancers.

## Introduction

A subset of patients with metastatic colorectal cancer (CRC) experiences long-term survival following curative-intent treatment with perioperative chemotherapy and surgical removal of primary tumors and limited liver metastases^1^. However, multiple randomized trials of adjuvant chemotherapy in this setting have failed to demonstrate a survival benefit^2–4^. Therefore, personalized approaches to identify patients most likely to benefit from perioperative systemic therapies are critically needed.

In this context, we previously defined three integrated molecular subtypes of mismatch repair-proficient (non-MSI) CRC liver metastases (CRCLM), which we designated as canonical, immune, and stromal subtypes^5^. Immune metastases exhibited favorable survival in association with immune infiltration and interferon and p53 pathway activation. By contrast, canonical metastases demonstrated adverse prognoses and overexpressed E2F/MYC and G2/M cell cycle proliferation pathways in an immune-depleted tumor microenvironment. Despite their overall poor prognosis, a subset of patients with canonical metastases exhibited favorable survival. We hypothesized that increased proliferation, in the context of tumor aneuploidy and immune exclusion, might contribute to the heterogeneous clinical outcomes of canonical metastases and CRCLM broadly.

Here, by leveraging three independent datasets (UCMC, MSKCC, and UK/New EPOC) of non-MSI CRCLM patients (*n* = 336, Methods and Supplementary Table 1), we investigated the prognostic significance of CIN70, a gene expression signature strongly correlated with proliferation in the context of tumor aneuploidy^6^. Despite its association with clinical outcomes across numerous datasets and cancer types, the role of CIN70 in CRCLM has not been explored. Finally, we explored the role of CIN70 as a potential biomarker for response to DNA-damaging therapies.

**Fig. 1:**
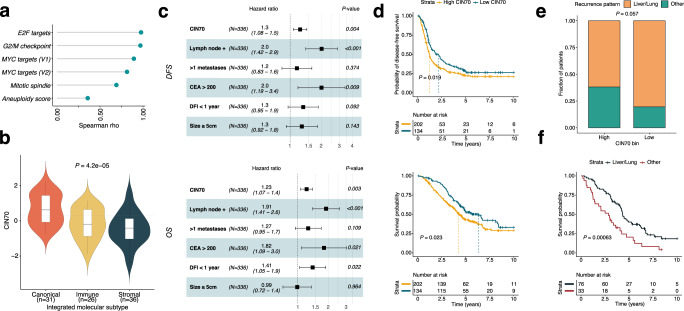
Prognostic value of CIN70 in CRCLM. **a** Spearman correlation of CIN70 score with Hallmark proliferation signatures and aneuploidy score; all correlations *P* < 0.05. **b** Comparison of CIN70 scores among CRCLM molecular subtypes; dashed line denotes median CIN70 score of all samples (*n* = 93); boxplot top and bottom edges represent the 1st and 3rd quartiles, respectively; the center line represents the median; whiskers extend to the farthest data points which do not represent outliers (within 1.5x the interquartile range); outliers are plotted as points above and below the box-and-whisker plot; Kruskal–Wallis test. **c** Forest plots of multivariable Cox proportional hazards models for DFS (top) and OS (bottom) of the pooled datasets (*n* = 336). Squares represent point estimates; bars represent 95% confidence intervals. Multivariable *P*-values and hazard ratios are displayed. CIN70 scores were evaluated as a continuous variable whereas other covariates with binary variables. CEA: carcinoembryonic antigen, DFI: disease-free interval between primary tumor and presentation of liver metastasis. **d** Kaplan–Meier curves of the pooled datasets (*n* = 336) for disease-free (upper panel) and overall survival (lower panel); log-rank test. High CIN70 defined as scores ≥40th percentile within each dataset; log-rank test; vertical dashed lines represent median survival time for each group. **e** Stacked bar plots showing the percentage of patients with liver/lung vs. other site metastatic recurrence following curative-intent treatment of CRCLM in the UCMC and MSKCC cohorts (*n* = 109); Fisher’s exact test. High CIN70 defined as scores ≥40th percentile within each dataset. **f** Kaplan–Meier curves showing overall survival for patients with metastatic recurrence to liver/lung vs. other sites; log-rank test.

First, we validated the molecular phenotype associated with the CIN70 signature in our dataset. We found that CIN70 was correlated with both cellular proliferation pathways and aneuploidy score in CRCLM, suggesting that CIN70 encompasses aspects of both features (Fig. 1a). Importantly, canonical metastases exhibited higher CIN70 scores as compared to immune and stromal metastases (Fig. 1b). In addition, higher CIN70 scores negatively correlated with numerous immune cell populations (Supplementary Fig. 1a).

Next, we explored the prognostic value of post-chemotherapy CIN70 in the setting of CRCLM. To maximize the power of our statistical analyses, we pooled all three CRCLM cohorts. We performed Z-score normalization of CIN70 scores within each cohort, which produced comparable CIN70 score distributions across the 3 cohorts (before normalization, all Kolmogorov-Smirnov test *P*-values < 1e−13; after normalization all *P-*values > 0.6, Supplementary Fig. 1b). No differences in DFS or OS were observed across the datasets (Supplementary Fig. 1c). Thus, we determined that it was appropriate to combine the datasets for subsequent analyses.

In a multivariable analysis of the pooled cohort that included established prognostic clinical and pathological features, the post-chemotherapy CIN70 score was independently prognostic of DFS (HR [per unit increase in Z-score]: 1.23 [95% CI: 1.07–1.40]) and OS (HR [per unit increase in Z-score]: 1.30 [95% CI: 1.08–1.50]) (Fig. 1c). Importantly, CIN70 remained significantly associated with inferior DFS (*P* < 2.2e−16) and OS (*P* < 2.2e−16) after controlling for tumor purity represented by the ESTIMATE score, suggesting that the CIN70 score was not confounded by differences in baseline tumor content.

Next, we set out to identify a CIN70 threshold that optimally risk-stratified patients in our study by testing each 10th percentile of CIN70 within each dataset in a leave-one-out cross validation analysis (Methods and Supplementary Fig. 1d). We determined that the 40th percentile of CIN70 by cohort was the optimal threshold based on the lowest average *P*-value DFS and OS (Fig. 1d).

In the UCMC and MSKCC cohorts, where patterns of failure data following initial treatment were available, metastases with high CIN70 scores were more likely to recur in organ sites beyond the liver and lung, compared to low-CIN70 metastases (*P* = 0.057, Fig. 1e). Notably, metastatic recurrence beyond the liver and lung was consistently associated with a higher risk of death following treatment (Fig. 1f). In addition, CIN70 scores were independent of established adverse clinical, pathological, and molecular prognostic factors (Supplementary Fig. 1e). These findings demonstrated that elevated CIN70 is associated with an increased propensity for metastatic dissemination and unfavorable survival in patients with CRCLM.

Notably, patients whose tumors exhibited a complete radiographic response (CR) to pre-operative chemotherapy harbored a lower post-chemotherapy CIN70 score versus patients whose tumors demonstrated a partial response (PR), stable disease (SD), or progressive disease (PD) (Supplementary Fig. 2a) in association with improved progression-free and overall survival (Supplementary Fig. 2b).

**Fig. 2:**
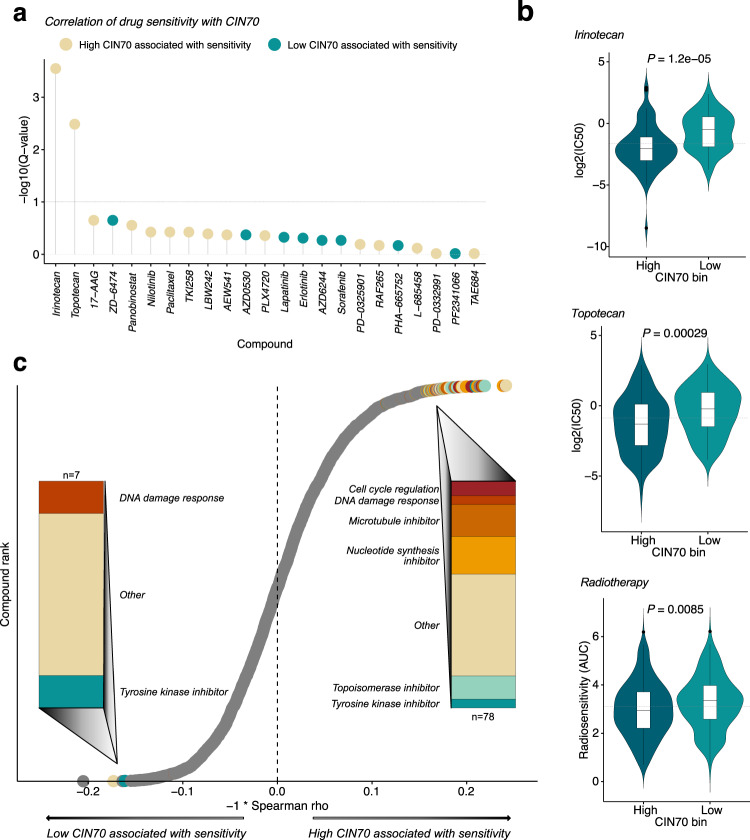
CIN70 predicts sensitivity to DNA-damaging agents. **a** Lollipop plot showing differences in the median log_2_(IC50) of 20 compounds tested on 246 CCLE carcinoma cell lines between high CIN70 (≥40th percentile) and low-CIN70 cell lines; color legend reflects median log_2_(IC50) of CIN70 high–low; dotted line denotes Q < 0.1. **b** Violin plots of irinotecan (*n* = 141), topotecan (*n* = 222), and radiosensitivity (*n* = 455) by CIN70 bin; boxplot top and bottom edges represent the 1st and 3rd quartiles, respectively; the center line represents the median; whiskers extend to the farthest data points which do not represent outliers (within 1.5x the interquartile range); outliers are plotted as points above and below the box-and-whisker plot; Wilcoxon test. Dashed lines indicate the median of all samples in the plot. **c** Spearman correlation of cell viability (log2[IC50]) with CIN70 for 4,686 drug compounds among 415 CCLE carcinoma cell lines; compounds with *Q* < 0.1 are colored by mechanism of action; bar plots represent fraction of significant compounds corresponding to each mechanism of action; dashed line indicates Spearman rho = 0.

Due to the association of post-chemotherapy CIN70 with radiographic response, we investigated whether the improved survival of CRCLM patients in the lowest quartile of CIN70 was related to the increased sensitivity of these metastases to DNA-damaging therapies, such as topoisomerase inhibitors, which are commonly utilized in the treatment of CRC. We examined the relationship between CIN70 values and sensitivity to topotecan and irinotecan as measured by IC50 in 246 carcinoma cell lines from the Cancer Cell Line Encyclopedia (CCLE) (Supplementary Table 2, Fig. 2a). Unexpectedly, we found that the carcinoma cell lines with high CIN70 levels (≥40th percentile) were most sensitive to topotecan and irinotecan (Fig. 2a). Similarly, high-CIN70 cell lines were sensitive to other DNA-damaging therapies, including ionizing radiation (Fig. 2b). By contrast, we observed no relationship between CIN70 and sensitivity to agents not considered to elicit DNA damage. We examined an additional 4,686 drug compounds in the DepMap database in which cell viability was measured after treatment in 415 carcinoma cell lines (Supplementary Tables 3-4). Drugs whose cell viability score was negatively correlated with CIN70, indicating increased sensitivity with higher CIN70, were enriched for DNA-damaging compounds such as topoisomerase, nucleotide synthesis, and microtubule inhibitors (Fig. 2c), consistent with evidence that chromosomal untangling and packaging is defective in chromosomally unstable cancer cells^7^.

**Fig. 3:**
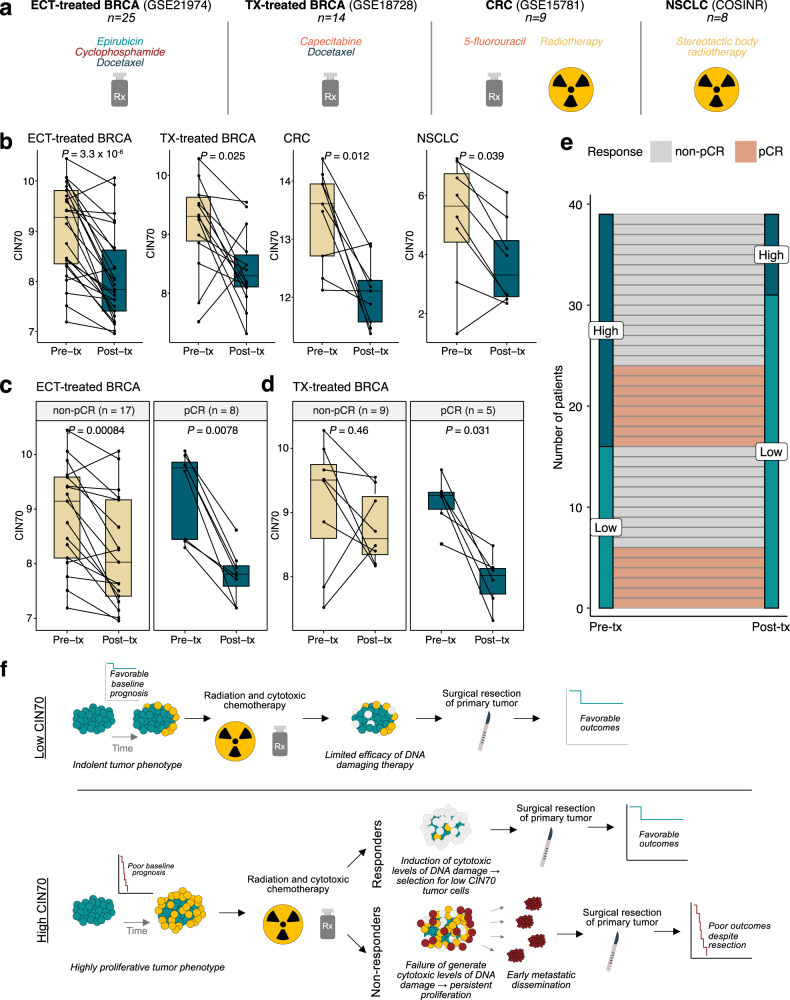
Changes in CIN70 following treatment are associated with improved clinical response. **a** Clinical cohorts containing pre- and post-treatment biopsies. **b** Boxplots showing CIN70 score changes between pre-treatment and post-treatment samples of four cohorts with matched pre-/post-treatment biopsies; boxplot top and bottom edges represent the 1st and 3rd quartiles, respectively; the center line represents the median; whiskers extend to the farthest data points which do not represent outliers (within 1.5x the interquartile range); outliers are plotted as points above and below the box-and-whisker plot; paired Wilcoxon test. Paired boxplots showing CIN70 score changes between pre-treatment and post-treatment samples in tumors that showed a pathologic complete response (pCR) vs. non-pCR in the (**c**) ECT-treated breast cancer and (**d**) TX-treated breast cancer cohorts; paired Wilcoxon test. **e** Alluvial plot showing changes in CIN70 bin from pre- to post-treatment samples in the combined ECT- and TX-treated cohorts (*n* = 39); CIN70 bins on both left and right of plot are defined using the 40th percentile breakpoint from pre-treatment samples determined separately within each cohort. **f** Proposed mechanism of observed response and outcomes among high versus low-CIN70 tumors.

Given that high CIN70 was associated with sensitivity to DNA-damaging agents in in vitro datasets and low post-chemotherapy CIN70 was associated with radiographic response and improved survival, we investigated whether DNA-damaging therapies deplete high-CIN70 cells within human tumors in association with favorable treatment responses. We tested this hypothesis in four independent clinical cohorts (*n* = 56) containing pre-treatment and post-treatment tumor biopsies (Fig. 3a). In all four datasets, we found a significant reduction in CIN70 following treatment (Fig. 3b). Importantly, after neoadjuvant chemotherapy for breast cancer, patients with pathologic complete responses (pCR) demonstrated a larger decrease in CIN70 than patients without pCRs (non-pCR) in both the ECT-treated (Fig. 3c) and TX-treated (Fig. 3d) breast cancer cohorts. Moreover, we observed that breast tumors with a low baseline level of CIN70 prior to treatment generally exhibited a stable or modest decrease in CIN70 following pre-operative chemotherapy. For breast cancers with initially high levels of CIN70, tumors exhibiting a pCR demonstrated a sharp decrease in CIN70 following therapy. By contrast, among tumors with a pre-treatment CIN70 score ≥40th percentile by dataset, those which had a CIN70 score that decreased below the original 40th percentile following treatment were significantly more likely to exhibit a pCR (*P* = 0.019, Fig. 3e). In a multivariable logistic regression of change in CIN70 and change in ESTIMATE score (a proxy for tumor purity), CIN70 remained an independent predictor of pCR (*P* = 0.0088), while ESTIMATE score did not (*P* = 0.83), suggesting that changes in CIN70 were not purely due to dilution of tumor content due to cell killing.

Interestingly, in the ECT-treated breast cancer cohort, CIN70 scores were greatest in triple-negative breast cancers (TNBC) relative to ER/PR+ and HER2+ breast cancers (Supplementary Fig. 3a), consistent with evidence for increased CIN in TNBCs^8^. While a reduction in CIN70 during neoadjuvant chemotherapy was observed across all subtypes of breast cancer (Supplementary Fig. 3b), this reduction was greatest within the TNBC subtype, suggesting that the higher baseline CIN70 in these tumors was associated with greater sensitivity to DNA-damaging therapy. This is consistent with the increased pCR rate in TNBC compared to non-TNBC after DNA-damaging neoadjuvant chemotherapy^9^.

Taken together, in the UK/New EPOC randomized trial, we observed that tumors with high levels of CIN70 exhibited inferior outcomes to those with scores less than the 40th percentile. As this cohort is composed of tumor samples after treatment with pre-operative chemotherapy, we propose that low-CIN70 CRCLMs represent a combination of two etiologies: (1) tumors with low baseline CIN70 and (2) tumors whose high pre-treatment CIN70 decreased in response to pre-operative chemotherapy. The reduction in CIN70 in the latter group is likely a consequence of the depletion of proliferative, high-CIN70 tumor cells. While these two groups have similar outcomes, we hypothesize the reasons for their improved survival are distinct. Tumors with inherently low-CIN70 scores likely display an indolent phenotype characterized by relatively low proliferation and CIN. In contrast, those whose CIN70 scores are reduced following cytotoxic therapy may represent patients who initially would have harbored tumors with an aggressive, proliferative phenotype, which was overcome by cytotoxic chemotherapy (Fig. 3f).

Previous studies have demonstrated that CIN is a prognostic factor in CRC^10,11^ and is associated with response to DNA-damaging therapies, such as taxanes in breast cancers^12^. In addition, a higher CIN70 score has been shown to be associated with worse prognosis across various cancer types^6^. However, the prognostic impact of CIN70 in the setting of colorectal liver oligometastases has not previously been described, and its relationship with treatment response to other DNA-damaging therapies such as radiotherapy has not been explored. In our study of 336 patients with CRCLM, low-CIN70 was associated with superior DFS and OS, along with favorable patterns of metastatic recurrence. Moreover, in vitro analyses revealed that high CIN70 correlated with sensitivity to DNA-damaging therapies, including topoisomerase inhibitors and radiotherapy. In patients receiving chemotherapy, large reductions in CIN70 translated to improved pathologic responses and predominantly occurred in patients with high pre-treatment CIN70. Collectively, these findings provide a potential opportunity to personalize treatment among patients with oligometastatic CRC. Considering the uncertain benefit of perioperative chemotherapy for resected CRCLM, CIN70 may serve as a biomarker of chemotherapy benefit. Finally, we also demonstrated that these observations are not limited to CRC; similar results were observed across pan-cancer cell lines and human breast and lung cancers.

An unanswered question is how to distinguish high-CIN70 tumors that are likely to respond to DNA-damaging therapies from those unlikely to benefit. Future multi-omic studies are needed to identify baseline clinicogenomic biomarkers which can complement CIN70 to further personalize therapeutic interventions, including DNA-derived measures of chromosomal instability, specific genomic alterations^13^, and multi-gene classifiers^14^. Moreover, it is important to validate these observations in prospective clinical trials to confirm whether CIN70 may serve as a clinical biomarker of response to cytotoxic chemotherapy and provide tailored treatments to patients across cancer types.

## Materials and methods

### Data sources

The UCMC dataset of 93 patients treated at The University of Chicago Medical Center (Chicago, IL) and NorthShore University Hospital was previously described^5^ and is available for download from the European Genome-Phenome Archive (https://ega-archive.org/studies/EGAS00001002945). No consent was required for the UCMC cohort due to the retrospective nature of the study. Patients in this cohort with colorectal adenocarcinoma underwent hepatic resection for limited liver metastases that presented either synchronously or metachronously (typically 1-5 lesions involving one or both lobes). 98% of patients received perioperative chemotherapy that were considered standard of care at the time of treatment. Patients predominantly received perioperative systemic therapy, consisting of 5-fluorouracil-based chemotherapy typically combined with oxaliplatin and/or irinotecan, curative-intent management of primary colorectal tumors, and partial hepatectomy of all visible liver metastases. Molecular subtype data was obtained from the prior publication^5^.

RNA-seq analysis was carried out by first aligning 75 bp length paired-end reads to the hg38 human reference genome using the *STAR* aligner version 2.6.1d^15^. The resulting .bam files were then sorted using *samtools* version 1.10^16^. The total reads per gene were counted using *htseq* in stranded mode with the Ensembl hg38 list of coding exons for each gene as a reference. A matrix of counts for each gene and sample was then generated. The R package *edgeR*^17^ was used to generate a table of logCPM values for each gene.

The MSKCC dataset^14^ of 96 patients with colorectal liver metastases was downloaded from the ArrayExpress website (https://www.ebi.ac.uk/arrayexpress/experiments/E-MTAB-1951/). No consent was required for the MSKCC cohort due to the retrospective nature of the study. Affymetrix .CEL files were processed using the software suit Analysis Power Tools (APT). The following command line was used: apt-probeset-summarize -rma -d chip.cdf -o output-dir –cel-files cel_list.txt. The APT software carried out background subtraction, RMA normalization, and summarization of all the probes in the probeset. All RMA output was then log2 transformed. As in the UCMC dataset, patients predominantly received 5-fluorouracil-based perioperative chemotherapy with oxaliplatin and/or irinotecan, curative-intent management of primary colorectal tumors, and partial hepatectomy of all visible liver metastases.

The UK/New EPOC dataset was downloaded from a privately accessed cBioPortal request following MTA approval by the Stratification in Colorectal Cancer (S:CORT) consortium. The study (ISRCTN 22944367) was approved by the South West Research Ethics Committee. Written informed consent was obtained from all patients prior to randomization.

Treatment protocols were administered according to the current standard of care as described above in the UCMC and MSKCC datasets. In the UK/New-EPOC cohort, ~50% of the patients also received perioperative cetuximab. Archival liver metastasis and primary tumor FFPE blocks from the New EPOC clinical trial were profiled using microarrays. Tumor material was identified on an adjacent hematoxylin and eosin–stained slide for macrodissection. Total RNA was extracted from sequential 5-mm sections using the Roche High Pure FFPE Extraction Kit (Roche Life Sciences) and amplified using the NuGen Ovation FFPE Amplification System v3 (NuGen). The amplified product was hybridized to the Almac Diagnostics XCEL array (Almac), a cDNA microarray-based technology optimized for archival FFPE tissue and analyzed using the Affymetrix Genechip 3000 7G scanner (Affymetrix). Quality control metrics relating to monitor image quality, in vitro transcription, hybridization to the array, and RNA degradation were assessed prior to uploading to the S:CORT server, where further quality control was performed. CEL files were downloaded and processed using the Affymetrix Array Power Tools (APT) as described above (https://www.thermofisher.com/us/en/home/life-science/microarray-analysis/microarray-analysis-partners-programs/affymetrix-developers-network/affymetrix-power-tools.html). 221 of 257 patients in the UK/New-EPOC cohort underwent surgery after perioperative chemotherapy of which 147 CRC liver metastases with matched primary tumors successfully underwent molecular analysis.

The normalized gene expression and drug sensitivity data (log_2_[IC50]) were downloaded from the Cancer Cell Line Encyclopedia (CCLE) website (see Supplementary Tables, https://portals.broadinstitute.org/ccle) and the DepMap Portal (see Supplementary Tables, *CCLE.primscreen.l2fc.csv* from https://depmap.org/portal/). Given the baseline differences in drug sensitivity based on tumor histology, we utilized the subset of cell lines denoted as carcinomas for analysis. Radiosensitivity data, defined by the area under the curve (AUC), were obtained from Yard et al.^18^.

The COSINR dataset is a cohort of patients from an institutional phase I clinical trial^19^ treated with metastatic non-small cell lung cancer treated with stereotactic body radiotherapy (SBRT) with sequential or concurrent immune checkpoint blockade (ipilimumab/ nivolumab). Biopsies from the same metastatic lesion were obtained prior to radiotherapy and within one week after completion of SBRT for patients treated on the sequential arm to assess the impact of SBRT prior to the administration of immunotherapy. Genomic and transcriptomic data for this cohort are available online at the European Genome-Phenome Archive (EGAS00001006212). We have obtained written informed consent for all patients in COSINR (NCT03223155) dataset and have obtained appropriate approval from the University of Chicago Institutional Review Board.

Normalized gene expression data for GSE21974, GSE18728, and GSE15781 were downloaded from the GEO data repository (https://www.ncbi.nlm.nih.gov/geo/). Breast cancer subtyping was determined based on current (at the time of publication) clinical guidelines using the provided sample metadata in GEO (ER+: ≥1% of cells positive by immunohistochemistry [IHC], PR+: ≥1% of cells positive by IHC; HER2+: 3+ expression by fluorescent in-situ hybridization).

### CIN70 score calculation

CIN70 score calculation was performed as previously described^6^ using the average of the normalized gene expression data of the 70 genes that constitute the signature. In the case of microarray datasets such as GSE21974, GS18728, GSE48277, GSE15781, MSKCC, and UK/New EPOC, the probe with the maximum RMA-normalized average signal for each gene in the signature was used for calculating CIN70. CIN70 scores were Z-score normalized within each dataset using the *scale* function in R.

### CIN70 score threshold identification

Leave-one-out cross validation (LOOCV) analysis was performed to determine an optimal threshold to define high CIN70. Candidate thresholds of the 10th to 90th percentile within each dataset in steps of 10 were analyzed. A Kaplan–Meier survival model of high versus low CIN70 was computed for DFS and OS of the patients with tumors harboring an CIN70 greater than or equal to each candidate threshold. This process was repeated *n* times, where *n* is the cohort size, leaving out one unique patient in each iteration. For each candidate threshold, the mean Log-rank p-value across the n subsets and mean difference in 12-month survival between the groups. The optimal threshold was determined by selecting the candidate threshold with the lowest mean *P*-value across the DFS and OS models.

### Aneuploidy score calculation

Arm-level somatic copy-number alterations (aSCNAs) were called using ASCETS^20^ on segmented copy-number data with the default parameters (log_2_ copy ratio threshold = ±0.2; arm alteration fraction threshold = 0.7; min breadth of coverage [BOC] = 0.5). Aneuploidy scores were calculated for each sample by computing the number of arms affected by aSCNAs (ASCETS call = AMP or DEL). However, panel data can result in insufficient BOC to make reliable aSCNA calls on some chromosome arms (ASCETS call = LOWCOV). Thus, we modified the score by dividing the number of arms with aSCNAs by the total number of arms with sufficient BOC in the sample (ASCETS call = AMP, DEL, NEUTRAL, or NC). The final scores represent the fraction of evaluable arms harboring aSCNAs in each sample.

### Hallmark pathways

Single-sample GSEA was performed on the UCMC cohort RNA-seq logCPM data and the MSKCC/UK normalized microarray expression data using the *gsva* package in R with the following parameters (method = “ssgsea”, kcdf = “Poisson”) on the set of Hallmark gene sets (h.all.v7.4.symbols.gmt).

### ESTIMATE score calculation

The Estimation of STromal and Immune cells in MAlignant Tumours using Expression data (ESTIMATE) algorithm^21^ was implemented using the R “*estimate*” package (https://bioinformatics.mdanderson.org/public-software/estimate/).

### MCPcounter cell population signature analysis

MCPcounter scores for each sample were calculated using the *MCPcounter* package in R on either the microarray data (MSKCC and UK cohorts) or on log_2_(CPM) values for RNA-seq datasets (UCMC cohort).

### Statistical analysis

All analyses were performed using R version 3.5.1. Data were analyzed Kruskal-Wallis, Wilcoxon, and Fisher’s exact tests as appropriate. Correlations between continuous variables were assessed using a Spearman correlation. In the case of matched data, a paired two-tailed Wilcoxon signed-rank test was used. All tests were two-tailed and *P* < 0.05 was considered significant. *P*-values were corrected for multiple comparisons using the Benjamini–Hochberg method for false discovery rate. Logistic regression was performed using the *glm* function in R using the “binomial” model family.

Progression-free survival was defined as the interval between the start of neoadjuvant chemotherapy and disease progression or death (event) or last follow-up (censor). Disease-free survival was defined as the interval between surgical resection of all visible disease and disease progression or death (event) or last follow-up (censor). Overall survival was defined as the interval between the end of treatment and death (event) or last follow-up (censor). Kaplan–Meier curves were generated using the *survminer* package in R; a log-rank test was used to compare survival between groups. Hazard ratios and confidence intervals were calculated using a Cox proportional hazards regression model.

### Reporting summary

Further information on research design is available in the Nature Research Reporting Summary linked to this article.

## Supplementary information

REPORTING SUMMARY

Supplementary Information

Supplementary Data 1-4

## Data Availability

The UCMC dataset of 93 patients treated at The University of Chicago Medical Center (Chicago, IL) and NorthShore University Hospital was previously described and is available for download from the European Genome-Phenome Archive (https://ega-archive.org/studies/EGAS00001002945). The MSKCC dataset of 96 patients with colorectal liver metastases was downloaded from the ArrayExpress website (https://www.ebi.ac.uk/arrayexpress/experiments/E-MTAB-1951/). The UK/New EPOC dataset was downloaded from a privately accessed cBioPortal request following MTA approval by the Stratification in Colorectal Cancer (S:CORT) consortium. The UK/New EPOC dataset is available from the authors upon reasonable request, with no restrictions to access or use of data. Please contact: enric.domingo@oncology.oc.ac.uk. The COSINR dataset: Genomic and transcriptomic data for this cohort are available online at the European Genome-Phenome Archive (EGAS00001006212). The GEO datasets: Normalized gene expression data for GSE21974, GSE18728, and GSE15781 were downloaded from the GEO data repository (https://www.ncbi.nlm.nih.gov/geo/).
