# Supplementary material for: A proliferative subtype of colorectal liver metastases exhibits hypersensitivity to cytotoxic chemotherapy
Source: NPJ Precis Oncol. 2022 Oct 14;6:72. doi: 10.1038/s41698-022-00318-z (PMC9568565; doi:10.1038/s41698-022-00318-z)
Supplement: Supplementary file 1 — REPORTING SUMMARY [file 41698_2022_318_MOESM1_ESM.pdf]

nature portfolio

Corresponding author(s): Sean P. Pitroda

Last updated by author(s): Sep 2, 2022

Reporting Summary

Nature Portfolio wishes to improve the reproducibility of the work that we publish. This form provides structure for consistency and transparency in reporting. For further information on Nature Portfolio policies, see our Editorial Policies and the Editorial Policy Checklist.

Statistics

For all statistical analyses, confirm that the following items are present in the figure legend, table legend, main text, or Methods section.

n/a	Confirmed	
☐	☒	The exact sample size (*n*) for each experimental group/condition, given as a discrete number and unit of measurement
☒	☐	A statement on whether measurements were taken from distinct samples or whether the same sample was measured repeatedly
☐	☒	The statistical test(s) used AND whether they are one- or two-sided<br>*Only common tests should be described solely by name; describe more complex techniques in the Methods section.*
☐	☒	A description of all covariates tested
☐	☒	A description of any assumptions or corrections, such as tests of normality and adjustment for multiple comparisons
☐	☒	A full description of the statistical parameters including central tendency (e.g. means) or other basic estimates (e.g. regression coefficient) AND variation (e.g. standard deviation) or associated estimates of uncertainty (e.g. confidence intervals)
☐	☒	For null hypothesis testing, the test statistic (e.g. *F*, *t*, *r*) with confidence intervals, effect sizes, degrees of freedom and *P* value noted<br>*Give P values as exact values whenever suitable.*
☒	☐	For Bayesian analysis, information on the choice of priors and Markov chain Monte Carlo settings
☒	☐	For hierarchical and complex designs, identification of the appropriate level for tests and full reporting of outcomes
☐	☒	Estimates of effect sizes (e.g. Cohen's *d*, Pearson's *r*), indicating how they were calculated

Our web collection on statistics for biologists contains articles on many of the points above.

Software and code

Policy information about availability of computer code

Data collection	No software was used to obtain data in this study.
Data analysis	RNA-seq analysis was carried out in the UCMC cohort using the STAR aligner version 2.6.1d. The resulting .bam files were sorted using samtools version 1.10; read counts were obtained using htseq; the edgeR R package was used to generate a table of logCPM values for each gene.<br><br>Arm-level somatic copy number alterations in the UCMC cohort were called using ASCETS v1.1.<br><br>Cell population signatures were calculated using the MCPcounter package in R. Single-sample GSEA was performed using the gsva package in R. Kaplan-Meier curves were generated using the survminer package in R. ESTIMATE scores were calculated on the logCPM or normalized array expression matrix in R. All statistical analyses were performed in R v4.2.0.

For manuscripts utilizing custom algorithms or software that are central to the research but not yet described in published literature, software must be made available to editors and reviewers. We strongly encourage code deposition in a community repository (e.g. GitHub). See the Nature Portfolio guidelines for submitting code & software for further information.

Data

Policy information about availability of data

All manuscripts must include a data availability statement. This statement should provide the following information, where applicable:

- Accession codes, unique identifiers, or web links for publicly available datasets
- A description of any restrictions on data availability
- For clinical datasets or third party data, please ensure that the statement adheres to our policy

All data and code were obtained from existing public sources with the following exceptions: (1) UK/New-EPOC genomic and clinical data required MTA approval from S:CORT consortium and (2) clinical data for the MSKCC cohort required MTA approval from MSKCC.

Human research participants

Policy information about studies involving human research participants and Sex and Gender in Research.

Reporting on sex and gender	Data on sex and gender were not collected for this study. Patients were included regardless of sex and gender.
Population characteristics	Patient characteristics of each cohort are described in the following manuscripts:<br>UCMC cohort: Pitroda et al, Nat Comms 2018<br>MSKCC cohort: Ito et al, PLOS One 2013<br>UK/New EPOC cohort Bridgewater et al, Lancet Oncology 2020<br>COSINR cohort Bestvina, et al, JTO 2022
Recruitment	Participant recruitment is described in the manuscripts cited above in the "Population characteristics" section.
Ethics oversight	This study was approved by institutional review boards at each respective institution. We have obtained informed consent for all patients in the UK/New EPOC trial (ISRCTN 22944367) and COSINR (NCT03223155) datasets.

Note that full information on the approval of the study protocol must also be provided in the manuscript.

Field-specific reporting

Please select the one below that is the best fit for your research. If you are not sure, read the appropriate sections before making your selection.

☒ Life sciences ☐ Behavioural & social sciences ☐ Ecological, evolutionary & environmental sciences

For a reference copy of the document with all sections, see nature.com/documents/nr-reporting-summary-flat.pdf

Life sciences study design

All studies must disclose on these points even when the disclosure is negative.

Sample size	No methods were used to predetermine sample size. We used data from several datasets: the retrospective UCMC cohort of 93 patients with CRCLM, the retrospective MSKCC cohort with 96 patients with CRCLM, and the 147-patient UK/New-EPOC randomized prospective clinical trial dataset. In addition, we utilized several outside cohorts for validation: GEO datasets GSE21974 (n=25), GSE18728 (n=14), and GSE15781 (n=9), and the institutional COSINR dataset (n=8, EGAS00001006212).
Data exclusions	No patients were excluded from analysis from the sample sizes noted above except in cases of missing data required for specific analyses. N's have been included throughout the manuscript to note these occasions.
Replication	We utilized 3 independent cohorts of patients with CRCLM (total n=336) to demonstrate the replicability of our results. We applied our findings in a dataset of more than 400 human pan-cancer cell lines. Furthermore, we used four datasets of human lung, rectal, and breast cancers (total n=56) to show the applicability of our results to other cancer types. Details of which results were successfully replicated can be found in the main text.
Randomization	In the UK/New EPOC dataset, adult patients with KRAS wild-type resectable/suboptimally resectable CRCLM and a WHO performance status of 0–2 were randomized assigned to receive chemotherapy (+/-) cetuximab before and after liver resection. In all other datasets, no randomization was performed as these were retrospective studies. In the COSINR dataset, without consideration of PD-L1 expression or TMB, patients with Eastern Cooperative Oncology Group (ECOG) 0/1 performance status, measurable disease defined by RECIST v1.1, no symptomatic CNS disease, and no autoimmune or immunodeficiency syndromes were randomized to SBRT to 2-4 metastatic sites with concurrent or sequential (within 7 days) immunotherapy.
Blinding	No blinding was performed.

Reporting for specific materials, systems and methods

We require information from authors about some types of materials, experimental systems and methods used in many studies. Here, indicate whether each material, system or method listed is relevant to your study. If you are not sure if a list item applies to your research, read the appropriate section before selecting a response.

Materials & experimental systems

n/a	Involved in the study
☒	☐ Antibodies
☒	☐ Eukaryotic cell lines
☒	☐ Palaeontology and archaeology
☒	☐ Animals and other organisms
☒	☐ Clinical data
☒	☐ Dual use research of concern

Methods

n/a	Involved in the study
☒	☐ ChIP-seq
☒	☐ Flow cytometry
☒	☐ MRI-based neuroimaging
