# Supplementary material for: A proliferative subtype of colorectal liver metastases exhibits hypersensitivity to cytotoxic chemotherapy
Source: NPJ Precis Oncol. 2022 Oct 14;6:72. doi: 10.1038/s41698-022-00318-z (PMC9568565; doi:10.1038/s41698-022-00318-z)
Supplement: Supplementary file 2 — Supplementary Information [file 41698_2022_318_MOESM2_ESM.pdf]

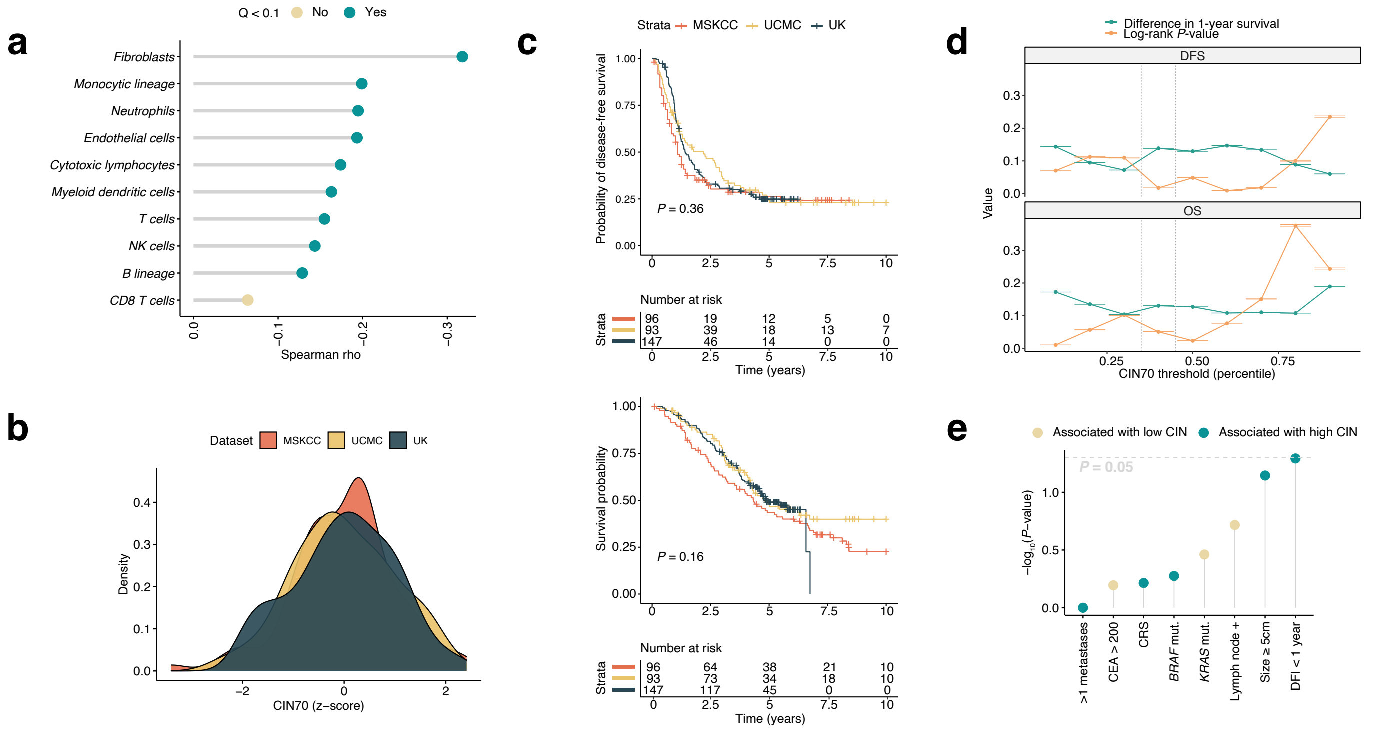

Supplementary Figure 1: a) Spearman correlation of MCPcounter signatures and CIN70 score in pooled CRCLM cohort. **b)** Density plots showing distributions of CIN70 Z-scores in each CRCLM dataset. **c)** Comparison of DFS (top) and OS (bottom) between the three CRCLM datasets; log-rank test. **d)** Line plot showing selection of optimal threshold at which to define high CIN70. Log-rank *P*-values and the difference in survival (high CIN70 – low CIN70) at each time point are plotted for each candidate threshold. Dotted lines surround the chosen threshold (lowest mean *P*-value across DFS and OS; 0.4). Dots represent point estimates; error bars represent 95% CI (n=336). **e)** Lollipop plots showing the associations of Clinical Risk Score (CRS) factors and pathogenic KRAS and BRAF mutations with low-CIN70 (<40th percentile) vs. high-CIN70 (≥40th percentile) tumors in the pooled UCMC, MSKCC, and UK datasets. CEA: carcinoembryonic antigen, DFI: disease-free interval between primary tumor and presentation of liver metastasis; Fisher's exact test. Dashed horizontal line corresponds to *P*=0.05.

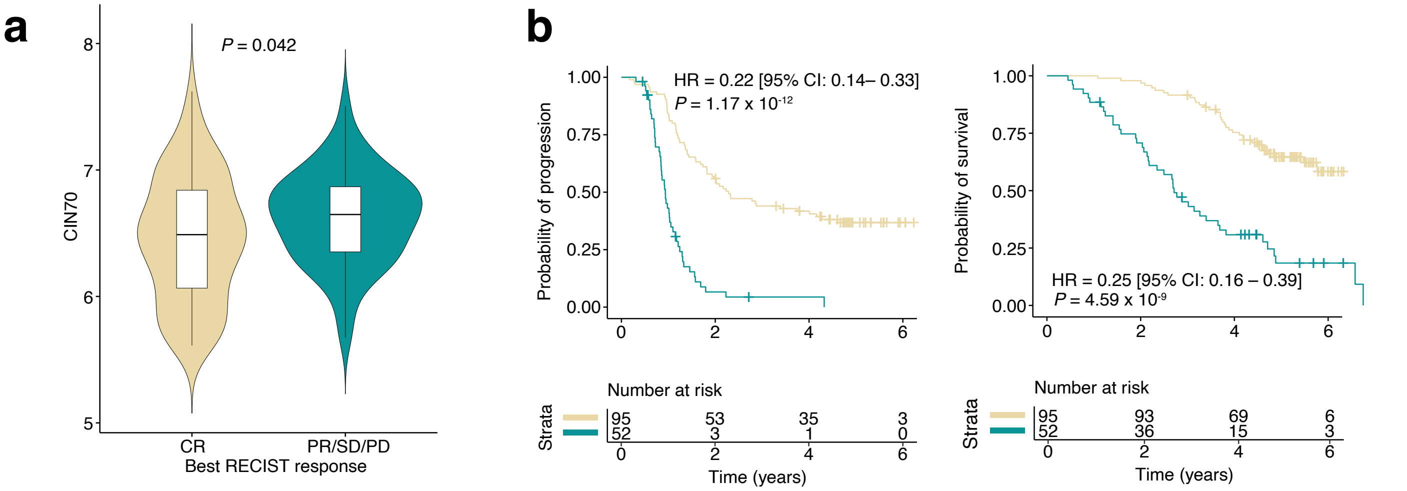

Supplementary Figure 2: a) Violin plot showing CIN70 scores in the UK/New-EPOC dataset for patients with CR vs. PR/SD/PD (n=147); Boxplot top and bottom edges represent the 1st and 3rd quartiles, respectively; the center line represents the median; whiskers extend to the farthest data points which do not represent outliers (within 1.5x the interquartile range); outliers are plotted as points above and below the box-and-whisker plot; Wilcoxon test. **b)** Kaplan-Meier curves of the UK/New-EPOC dataset (n=147) for progression-free survival (left) and overall survival (right) based on response to pre-operative chemotherapy split by radiographic RECIST complete response (CR) vs. partial response (PR)/stable disease (SD)/progressive disease (PD); log-rank test.

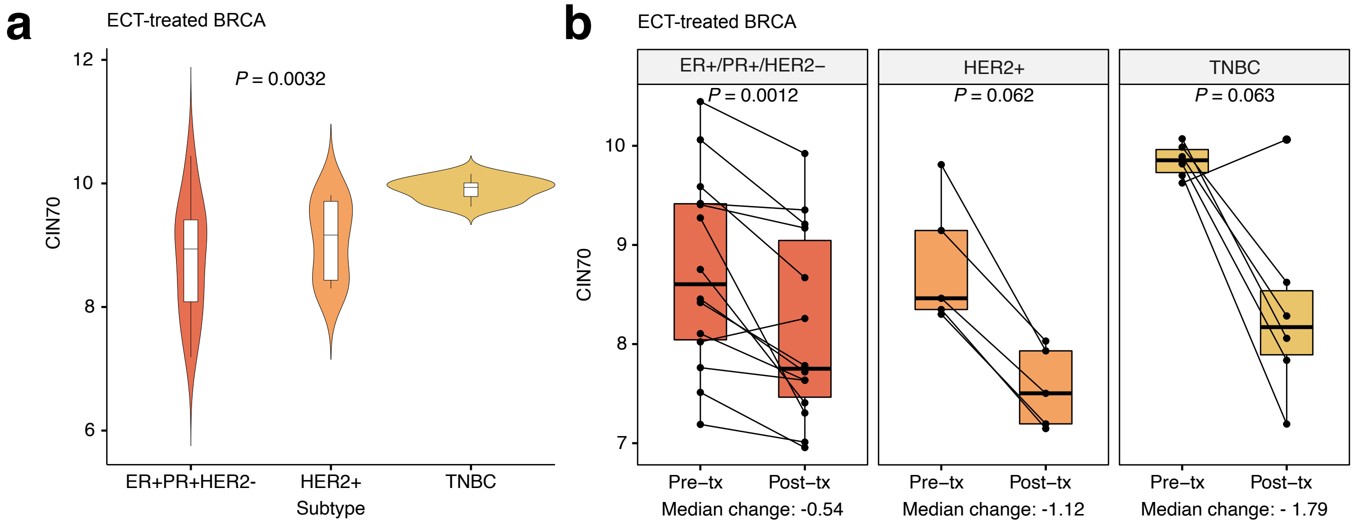

Supplementary Figure 3: a) Violin plot of pre-treatment CIN70 scores by breast cancer subtype in the ECT-treated BRCA cohort (n=32); Boxplot top and bottom edges represent the 1st and 3rd quartiles, respectively; the center line represents the median; whiskers extend to the farthest data points which do not represent outliers (within 1.5x the interquartile range); outliers are plotted as points above and below the box-and-whisker plot; Kruskal-Wallis test. **b)** Boxplots of CIN70 score change by breast cancer subtype in the ECT-treated BRCA cohort; boxplot top and bottom edges represent the 1st and 3rd quartiles, respectively; the center line represents the median; whiskers extend to the farthest data points which do not represent outliers (within 1.5x the interquartile range); outliers are plotted as points above and below the box-and-whisker plot; paired Wilcoxon test.
